# The first two months of the COVID-19 pandemic in Bosnia and Herzegovina: Single-center experience

**DOI:** 10.17305/bjbms.2020.4838

**Published:** 2020-08

**Authors:** Jurica Arapović, Siniša Skočibušić

**Affiliations:** 1 Department of Infectious Diseases, University Clinical Hospital Mostar, Mostar, Bosnia and Herzegovina; 2 Faculty of Medicine, University of Mostar, Mostar, Bosnia and Herzegovina

**Keywords:** COVID-19, SARS-CoV-2, epidemiology, mortality, Bosnia and Herzegovina

## Abstract

The novel coronavirus disease 2019 (COVID-19) pandemic caused by severe acute respiratory syndrome coronavirus 2 (SARS-CoV-2) is still progressing and has been recorded in more than 210 countries and territories worldwide. In Bosnia and Herzegovina, the first cases of COVID-19 were detected on March 5, 2020 in the entity of the Republic of Srpska and on March 9, 2020 in the entity of the Federation of Bosnia and Herzegovina. By May 16, 2020, more than 2,200 COVID-19 cases had been recorded in both entities, with a mortality rate of 5.8% (131 of 2,231 cases). The aim of this ongoing study is to present the current epidemiological and sociodemographic parameters of 380 COVID-19 patients diagnosed at the University Clinical Hospital Mostar (UCH Mostar) during the first two months of the COVID-19 pandemic. Of those 380 patients, 60 (15.8%) required hospitalization. The mortality rate was 5% (19/380). The highest mortality rate (15.2%, 12/79) was recorded in the patients aged ≥65 years. In addition to this single-center experience of the ongoing COVID-19 pandemic, we discuss the epidemiological measures imposed in Bosnia and Herzegovina, with an emphasis on the restrictive measures. The COVID-19 pandemic is still ongoing in Bosnia and Herzegovina.

## INTRODUCTION

At the end of 2019, China reported the first cases of a severe acute respiratory syndrome (SARS) like illness caused by novel coronavirus type 2 (2019-nCoV, later classified as SARS-CoV-2) in Wuhan. On January 30, 2020, the World Health Organization (WHO) declared the outbreak of SARS-CoV-2 infection in China a Public Health Emergency of International Concern [1,2]. Since then, this epidemic has spread worldwide with a mortality rate of 0.86%–7.71% in China [3] despite the fact that local and federal governments in China introduced the largest quarantine in the world ever. The quarantine zone across Hubei province included 57 million people, thus directly limiting the spread of the virus to the rest of China and neighboring countries [4]. This action was accompanied by numerous criticisms from the world health public authorities but was complimented by the WHO. On March 11, 2020, the WHO classified the spread of COVID-19 as pandemic [5]. It is now clear that after the SARS outbreak and 2009 H1N1 pandemic, the COVID-19 pandemic is the most emergent pandemic of the third millennium [6]. Immediately after the COVID-19 pandemic began in China, the global epidemic situation worsened and consequently transferred primarily to Europe. A devastating scenario of COVID-19 occurred primarily in Italy with a high spreading capacity to other European countries and to Southeastern Europe [7]. By May 16, 2020, more than 4,500,000 cases of COVID-19 with more than 300,000 deaths had been recorded worldwide [8]. In Bosnia and Herzegovina, the first imported cases of COVID-19 occurred on March 5, 2020 in the Republic of Srpska and on March 9, 2020 in the Federation of Bosnia and Herzegovina [7]. These first cases were mainly imported from Italy and afterward from other European countries where the pandemic was very active [7]. The first case of COVID-19 in Mostar was identified on March 16, 2020, and resulted in the spread of the pandemic to southern Bosnia and Herzegovina in the Herzegovina region. According to the WHO report, 18 cases were recorded on March 16, 2020 in Bosnia and Herzegovina that may have been locally transmitted [9]. On April 9, 2020, one month after COVID-19 was first detected in Bosnia and Herzegovina, the WHO classified Bosnia and Herzegovina as a country with potential for community transmission after 816 COVID-19 cases and 35 deaths had been reported [10].

In this short report, we present the results of the ongoing COVID-19 pandemic in southern Bosnia and Herzegovina, compiled by three counties gravitating to the UCH Mostar. Furthermore, we discuss how the restrictive preventive measures were influenced by the spreading of the COVID-19 pandemic in Bosnia Herzegovina.

## MATERIALS AND METHODS

This cross-sectional observational study included 380 subjects with COVID-19 who were diagnosed at the UCH Mostar during the period between March 16, 2020 and May 16, 2020. Among them, 58 patients were hospitalized at the UCH Mostar, and two patients were hospitalized at the General Hospital Livno (GH Livno). In addition to positive epidemiological data and clinical examination, the final diagnosis was confirmed by real-time reverse transcriptase polymerase chain reaction (rRT-PCR) for SARS-CoV-2 on respiratory samples (nasopharyngeal and/or oropharyngeal swabs). One or more of the following criteria were for necessary for admission: aged 65 or older, high comorbidity risk (e.g., diabetes, hypertension, overweight, immunosuppression, etc.), lower respiratory tract symptoms (cough or difficulty breathing or infiltrates on chest X-ray), and low peripheral oxygenation levels (≤92%) [11].

### Ethical statement

All procedures followed were in accordance with the ethical standards laid down in the 1964 Declaration of Helsinki and its later amendments. Because this was a retrospective database analysis, informed consent was not required, and any potentially identifying patient information was omitted.

### Statistical analysis

We analyzed the results using IBM SPSS Statistics for Windows, Version 23.0 (IBM Corp, Armonk, NY), and Microsoft Excel (365). The results are presented as absolute numbers (n) and percentages (%). The relationship between variables was determined with the Chi-square test (χ^2^ test). Categorical variables were assessed using Fisher’s exact test. All tests were two-tailed, and values of *p* < 0.05 were considered statistically significant.

## RESULTS

During the first 60 days of the COVID-19 pandemic, we analyzed 380 patients with COVID-19 with a median age of 47.5 years (range, 1 to 98 years).

Thirty-six (9.5%) patients were under 18 years old, 265 (69.7%) were 18 to 64 years old, and 79 (20.8%) were 65 or older. There was no statistical difference among patients with COVID-19 regarding age or gender distribution (Chi-square statistic = 5.519; p = 0.238) (Table 1).

**TABLE 1:**
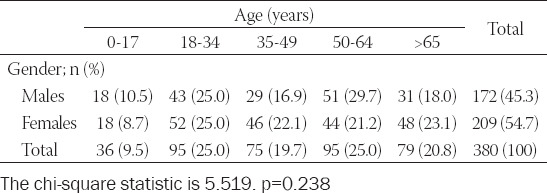
Gender and age distribution of the COVID-19 patients

During the first 60 days of the COVID-19 pandemic, 19 of the 380 patients died, resulting in a mortality rate of 5.0% (95% CI, 0.026–0.070). One patient died at GH Livno, two died at home, and 14 patients died at the UCH Mostar. The median age of the patients who died was 75 years (range, 52 to 98 years). The mortality rate among older patients (≥65 years) was 15.2% compared to 7.4% among 50 to 64 year-old patients (Table 2). Furthermore, all patients ≥65 years who died had at least one or more chronic comorbidities. Ten of them were males and seven were females (Fisher’s exact test, p = 0.320). We also observed 23 (6.1%) health care workers (HCWs) with COVID-19 (95% CI, 0.0406–0.0892).

**TABLE 2:**
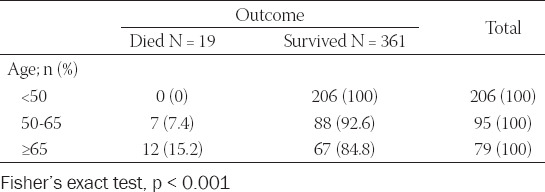
Age distribution and outcome of the COVID-19 patients

Of the 380 patients, 60 (15.8%) were hospitalized, and 54 (90%) of the hospitalized patients presented with pneumonia. The median age of the hospitalized patients with COVID-19 was 66.5 years (range, 20 to 89 years). Among the hospitalized patients with COVID-19, 20 (33.3%) were in the 50–64 year age group and 32 (53.3%) were 65 or older (Fisher’s exact test, *p* < 0.001). Additionally, 28 (46.7%) of the hospitalized patients required intensive care unit (ICU) treatment, and 20 (33.3%) were mechanically ventilated (Table 3). Notably, 7 (35%) patients survived after receiving mechanical ventilation support whereas 13 (65%) patients died (Fisher’s exact test, *p* < 0.001) (Table 4). All patients treated in the ICU had severe bilateral pneumonia and developed acute respiratory distress syndrome (ARDS).

**TABLE 3:**
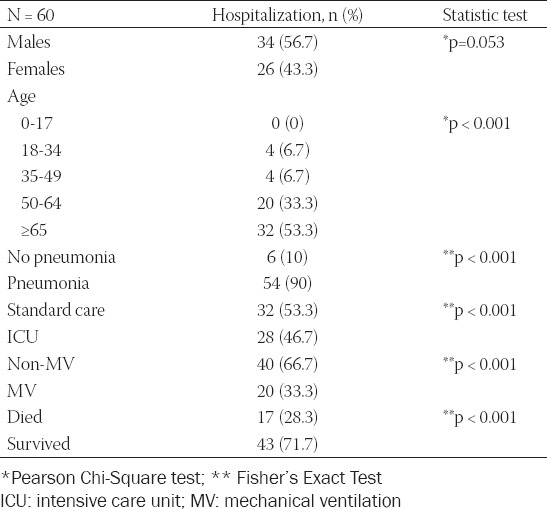
Analysis of patients hospitalized with COVID-19

**TABLE 4:**
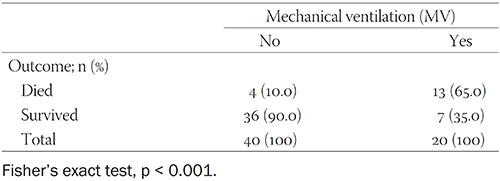
Survival rate of mechanically ventilated patients with COVID-19

## DISCUSSION

In this study, we report our preliminary results on the ongoing COVID-19 pandemic in three counties of the southern part of Bosnia and Herzegovina gravitating to the UCH Mostar. The mortality rate was 5% (19/380), which is slightly higher rate than in recently published results in Southeast European countries [7]. Many of the people who died from COVID-19 succumbed to severe pneumonia, which takes hold as the immune system is weakened from fighting the virus [3]. The mean age of the hospitalized patients was 66.5 years, and the mean age of patients who died from COVID-19 was 75 years, indicating that COVID-19 was the most severe in the elderly [12]. Notably, 35% (7/20) of the mechanically ventilated patients survived from severe pneumonia with ARDS. This is in accordance with previously published results showing that the mortality rate of mechanically ventilated COVID-19 patients ranged from 50–100% [13-16].

With the appearance of the first cases of COVID-19 in Bosnia and Herzegovina, governments of both entities declared the implementation of restrictive preventive measures on March 16, 2020 with several crucial restrictions (e.g., movement restrictions for those younger than 18 and older than 65; closure of national borders and curfews; closure of preschools, school institutions, universities, all public gatherings, and ordered cafes, bars, restaurants, and cultural institutions; public and city transport ban; comprehensive patient care reorganization in the public health system, etc.). Similar measures including curfews were implemented in all eastern European countries [7]. However, a recent study suggests that social distancing, hand wash hygiene, respiratory hygiene, and wearing masks, and gloves could help prevent the spread of COVID-19 [17].

It has been recently shown that restriction measures could help control the COVID-19 pandemic [17-19]. During the first 60 days in Bosnia and Herzegovina, the COVID-19 pandemic resulted in 29 deaths per million inhabitants, which was in accordance with the death rates in Serbia (22.9), Croatia (12.4), and N. Macedonia (43.2) but significantly lower than rates in Western European countries, e.g. Italy (165.8) or Spain (157) (Table 5). This short analysis indicated that that the promptly introduced epidemiological restriction measures and continuous monitoring by public health services resulted in better control of the pandemic in Southeastern European countries compared to some Western and high-income European countries.

**TABLE 5:**
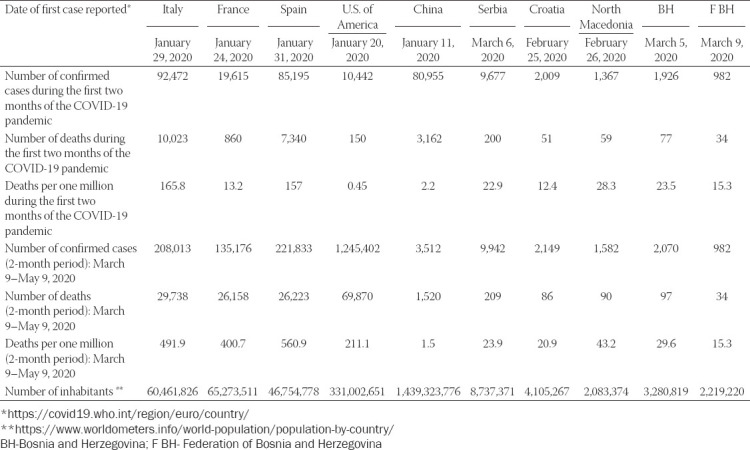
The effect of restrictive preventive measures on the mortality rate in the COVID-19 pandemic in Bosnia and Herzegovina

We also assume that the restrictive preventive measures established by the government of the Federation of Bosnia and Herzegovina for at least six weeks (March 16 to April 30, 2020) resulted in favorable outcomes of the pandemic in the Federation of Bosnia and Herzegovina (15.3 per million). According to the demographic statistical data, 2.20 million people live in the Federation of Bosnia and Herzegovina, with children, the middle-aged population, and the elderly comprising 21%, 66%, and 13% of the population, respectively. Interestingly, we observed that 20.8% of COVID-19 patients were elderly, which was more than expected proportionally for this age group, whereas a significantly lower number of children presented with COVID-19 compared to the demographic distribution of the pediatric age group (9.7% vs 21%). Significant movement may have occurred among middle-aged people, particularly asymptomatic individuals, which would have resulted in transmission of the infection to members of families at home [20]. The restrictive preventive measures were also implemented among school and preschool children by March 13, 2020 in Federation of Bosnia and Herzegovina. The relatively lower number of deaths suggests that these measures contributed significantly to a better response to the COVID-19 pandemic in this region.

Notably, 84.2% of COVID-19 patients presented with mild symptoms or were asymptomatic. All these patients were continuously monitored by general practice physicians and epidemiologists during at-home isolation. The second finding based on our good practices was the early testing of patients suspicious for COVID-19. In Bosnia and Herzegovina, preparedness and molecular screening of suspected subjects for imported COVID-19 resulted in the early detection and epidemiological surveillance of COVID-19 patients. We completely agree with the lesson learned by Italian colleagues that the early molecular detection of COVID-19 results in better monitoring of patients in at-home isolation [21]. Similarly, we also learned that nasopharyngeal and oropharyngeal swab testing should be performed at home by mobile and specialized microbiology teams with adequate personal protective equipment.

A limitation of this study is that it was a single-center experience, although it combines two hospitals and three larger administrative territories in Bosnia and Herzegovina. Thus, multi-centric studies should result in a more realistic view of the COVID-19 pandemic in Bosnia and Herzegovina, and upcoming studies should point out the significant clinical and therapeutic features of COVID-19 in Bosnia and Herzegovina.

## CONCLUSION

Here we examined the COVID-19 pandemic from a single-center point of view. Our results show that elderly people have a significant risk for COVID-19 and complications of the disease, with a high fatality rate, as observed worldwide. The national strategy led by the prompt epidemiological measures in Bosnia and Herzegovina during the COVID-19 pandemic resulted in a relatively small number of patients and deaths compared to high-income European countries affected by the COVID-19 pandemic. The second key message is a focus on the early testing of suspicious patients, which was a crucial preventive measure in the detection of COVID-19, and the subsequent proper isolation of COVID-19 patients at home. This pandemic is still ongoing in Bosnia and Herzegovina.
